# Targeting Hepatocellular Carcinoma: Schisandrin A Triggers Mitochondrial Disruption and Ferroptosis

**DOI:** 10.1111/cbdd.70010

**Published:** 2024-12-12

**Authors:** Lin‐wei He, Chang‐jie Lin, Lin‐jun Zhuang, Yi‐hui Sun, Ye‐cheng Li, Zhen‐yu Ye

**Affiliations:** ^1^ Department of General Surgery The Second Affiliated Hospital of Soochow University Souzhou Jiangsu China

**Keywords:** AMPK/mTOR pathway, apoptosis, ferroptosis, hepatocellular carcinoma, mitochondrial dysfunction, reactive oxygen species, Schisandrin A

## Abstract

The main focus of this research was to examine SchA's role in the hepatocellular carcinoma (HCC) development. LO2 and Huh7 cell viability were assessed using the MTT assay. The experiments included flow cytometry, colony formation, transwell, wound healing, and immunofluorescence assays to evaluate apoptosis levels, cells colony‐forming ability, ROS levels, invasion and migration ability, and mitochondrial membrane potential. Biochemical kits was utilized for checking the ATP, mitochondrial DNA, MDA, GSH, and Fe^2+^ levels in the Huh7 cells, and western blot for measuring the ferroptosis and AMPK/mTOR related‐protein expression levels. The MTT assay demonstrated that SchA significantly reduced the vitality of Huh7 cells ranging from 10 to 50 μM, whereas it exhibited no discernible impact on LO2 cells. Additionally, SchA significantly inhibited colony‐forming ability, invasion ability, and migration ability within the concentration range of 10 to 50 μM, with a reduction of 68% in colony formation at 50 μM. SchA also induced apoptosis in a dose‐dependent manner. Moreover, SchA was observed to significantly elevate ROS levels dose‐dependently, down‐regulate mitochondrial membrane potential (JC‐1) at 20 and 50 μM, and reduce the levels of ATP and mtDNA dose‐dependently. Various concentrations of SchA resulted in a notable elevation in MDA and Fe^2+^ levels as well as ACSL4 protein expression, accompanied by a reduction in GSH level and the protein expression of GPX4 and SLC7A11. Furthermore, SchA induced the activation of the AMPK/mTOR pathway in Huh7 cells, as evidenced by the increased phosphorylation level of AMPK and decreased phosphorylation level of mTOR. SchA might inhibit the progress of HCC through mitochondrial ferroptosis and dysfunction mediated by AMPK/mTOR pathway.

## Graphical abstract

This diagram illustrates the proposed mechanism by which Schisandrin A (SchA) affects hepatocellular carcinoma (HCC) cells. SchA induces reactive oxygen species (ROS) generation in mitochondria, leading to oxidative stress and mitochondrial dysfunction. This cascade activates AMP‐activated protein kinase (AMPK), which inhibits the mTOR pathway, resulting in decreased cell proliferation and increased apoptosis. Furthermore, the elevated ROS levels cause ferroptosis. This dual action of inducing apoptosis and ferroptosis effectively inhibits the migration and invasion of HCC cells, highlighting SchA's potential as a therapeutic agent against HCC.

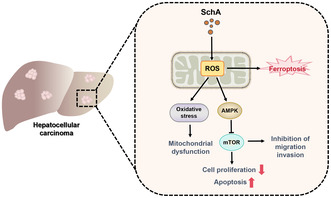

**Abbreviations**
FITC: fluorescein isothiocyanate
HCC: hepatocellular carcinoma
ROS: reactive oxygen species
SchA: Schisandrin A
SD: standard deviation

## 1 Introduction

As per the 2020 Global Cancer Statistics Report published by WHO, hepatocellular carcinoma (HCC) is a widespread malignant tumor. It ranks sixth in new cancer incidence and third in mortality rate at 8.3% (Zhu et al. 2023). Persistent liver damage caused by liver diseases is significantly linked to the HCC etiology. Notably, external causes like hepatitis B, chronic alcohol consumption, and exposure to aflatoxin constitutes 80% of liver diseases, making them paramount in the liver cancer development (Zhang et al. 2023). Systematic molecular targeted therapy and immune checkpoint suppression have progressively become HCC's first‐line and second‐line treatment methods (Faivre, Rimassa, and Finn 2020). Nevertheless, these treatments therapies failed to notably enhance HCC sufferers' median survival time. Furthermore, the issues of drug toxicity and resistance cannot be overlooked. Accordingly, the search for new drugs that are green, effective and safe is of utmost urgency.

Featured with immoderate lipid peroxidation and succeeding plasma membrane rupture, ferroptosis constitutes a form of iron‐dependent cell death (Tang et al. 2021). Since it was described in 2012 (Dixon et al. 2012), reports have highlighted the treatment potential of ferroptosis in treating diverse tumors (Stockwell, Jiang, and Gu 2020). For example, a study by Wu et al. (2022) provides a comprehensive analysis of how a ferroptosis‐related gene model can be used to predict outcomes in TNBC, potentially offering new avenues for personalized medicine and targeted therapies in this challenging subtype of breast cancer. In addition, the mitochondrial membrane potential loss also contacts with the early phase of ferroptosis. Mitochondrial dysfunction is related to numerous cell events with defects, comprising the reactive oxygen species (ROS) production. Commonly, ferroptosis is viewed as cell death that banks on ROS. In iron deficiency anemia, mitochondria affect the redox imbalance (Jelinek et al. 2018; Neitemeier et al. 2017). Besides, ferroptosis is a distinct non‐apoptotic cell death pattern, with numerous connections of apoptosis with ferroptosis delineated.

In recent years, people are increasingly interested in developing new drugs using natural products (Yoshimura et al. 2021; Zhu et al. 2019). Schisandrin A (SchA), called deoxyschizandrin also, emerges as among the most bioactive lignans separated from the fruit of *Schisandra chinensis* (Turcz.) Baill (Szopa, Ekiert, and Ekiert 2017). As stated by pharmacological researches previously, SchA possesses assorted biological activity characteristics including anti‐inflammatory, antioxidant, and antiviral effects (Kwon et al. 2018; Guo et al. 2016). However, it was later found that SchA also had anti‐tumor, anti‐allergic and anti‐fibrosis effects (Zhu et al. 2021; Moon, Jeong, and Kim 2012; Park and Yoon 2020). In the past 10 years, the research on the potential anti‐tumor effect of SchA has gradually increased, such as non‐small cell lung cancer, breast cancer, colon cancer, and esophageal cancer (Kwon et al. 2018; Yan and Guo 2020; Xian, Feng, and Zhang 2019). A study found that STA reduced the liver cancer cell (Hep3B and HCCLM3) proliferation and migration in vitro. It modulates glucose metabolism in Hep3B cells, decreasing the D‐glucose and lactate production. Additionally, subcutaneous tumor formation assays in nude mice proved that STA interference blocked the HCC tumor growth in vivo (Szopa, Ekiert, and Ekiert 2017). However, the mechanism of action of SchA on liver cancer is still insufficient. Therefore, we added the study of ferroptosis and AMPK/mTOR, which may provide reference for the clinical implementation of SchA in HCC treatment.

## 2 Materials and Methods

### 2.1 Cell Culture and Drug Preparation

**FIGURE 1:**
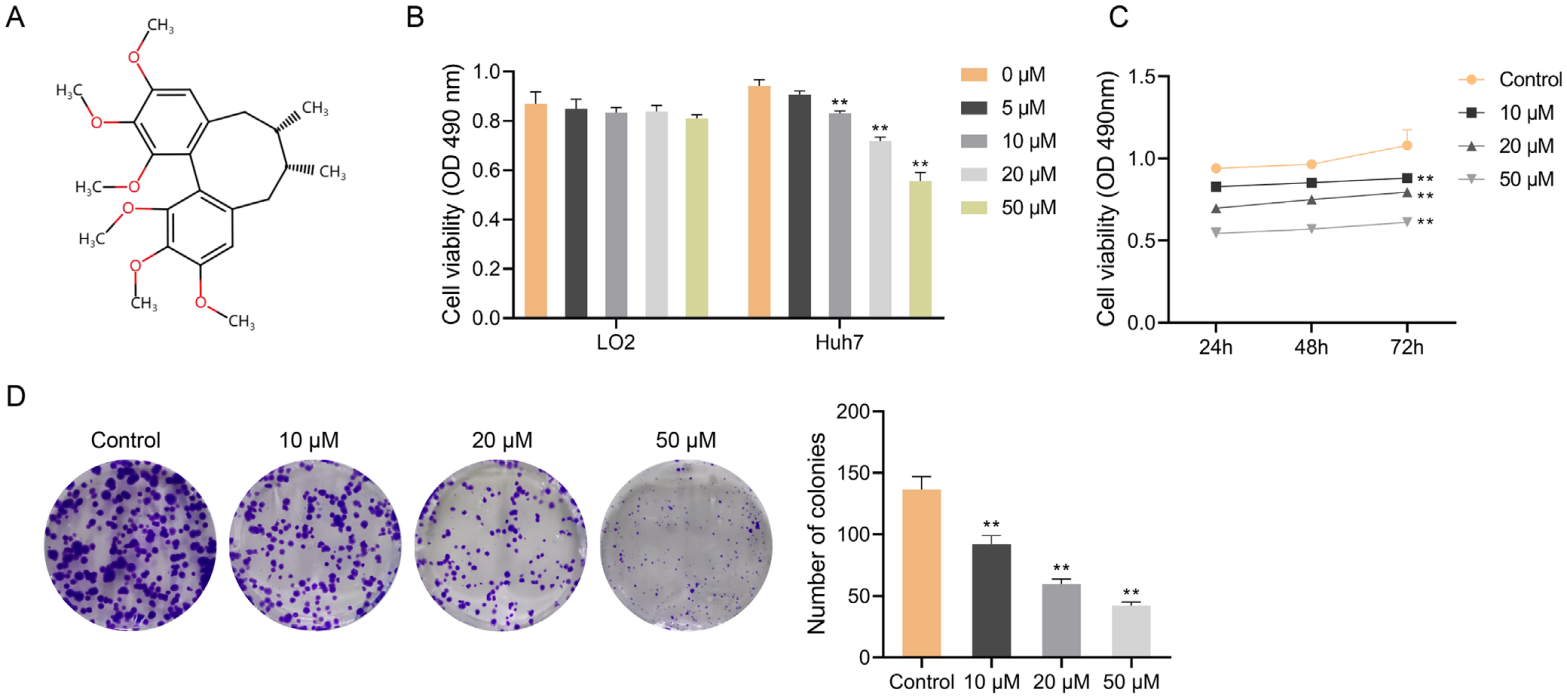
Schisandrin A inhibits the activity of hepatocellular carcinoma Huh7 cells. (A) Chemical structure of Schisandrin A. (B) MTT assay was used to detect the viability of LO2 and Huh7 cells treated with 0, 5, 10, 20, and 50 μM Schisandrin A at 24 h. (C) MTT assay was used to detect the viability of Huh7 cells treated with 0, 10, 20, and 50 μM Schisandrin A at 24, 48 and 72 h. (D) Cell cloning assay was used to test the cloning ability of Huh7 cells. **p < 0.01.

SchA (the chemical structure was displayed in Figure 1A) was procured from Chengdu Must Bio‐Technology Co. Ltd. (cat. no. 19092908). LO2 cells were offered via the Shanghai Cell Bank of the Chinese Academy of Sciences (Shanghai, China). HCC cells Huh7 were provided by the American Type Culture Collection (ATCC, USA). The cell culture was performed in DMEM medium (Thermo Fisher Scientific, Inc.) supplemented with 10% FBS (Thermo Fisher Scientific, Inc.). Incubation of the cells was conducted in a incubator at 37°C with 5% CO_2_.

### 2.2 MTT Assay

LO2 and Huh7 cells (5 × 10^3^ cells/well) were seeded into 96‐well plates. After seeding, the cells were treated with various concentrations of SchA (0, 5, 10, 20, and 50 μM) for 24 h. Subsequently, 10 μL of MTT solution was added to each well for additional 4 h. Afterwards, 150 μL of DMSO was added to dissolve the crystals. Measurement of absorbance at 490 nm was performed through a microplate reader (Bio‐Rad).

### 2.3 Colony Forming Assay

In 6‐well plates, Huh7 cells in the logarithmic growth phase were cultured in a complete medium with 30% FBS. Upon cell adhesion, medium containing 0, 10, 20, and 50 μM SchA was added. Change the suspension every 3 days and observe the cell state. Termination of the culture occurred when the cell count in most clones exceeded 50. Cells in each well were treated with 4% paraformaldehyde (1 mL) and fixed at 4°C. Following 60 min, they were stained utilizing crystal violet staining solution. The clone size was observed under the microscope and the photos were captured.

### 2.4 Cell Apoptosis Assay

Huh7 cells were inoculated with 4 × 10^4^ cells per well into a 6‐well plate. Cells were collected after 24 h of intervention with 0, 10, 20, and 50 μM SchA and then resuspended in the binding buffer. Next, the intervening cells received staining in a darkroom for 15 min by fluorescein isothiocyanate (FITC)‐labeled Annexin V (Biolegend, 0.45 μg/mL) and propidium iodide (5 μg/mL, Biolegend, Invitrogen). Then, the apoptosis rate within 1 h was tested via flow cytometry as described previously (Yang et al. 2023). Lastly, the green fluorescence was displayed by the annexin V‐FITC, and the red fluorescence by PI.

### 2.5 ROS Detection

Huh7 cell suspension was inoculated into a plate with 6 wells (2 × 10^5^ cells per well). Cells were exposed to SchA (0, 10, 20, and 50 μM) at 37°C, and 24 h later, the cell culture medium was offered the ROS DCFH‐DA probe at a ratio of 1:1000. Then, the cells were cultured at 37°C for 20 min and received washing twice utilizing PBS. Ultimately, a FACScanto II flow cytometer (BD Biosciences, Franklin Lakes, NJ, USA) was recruited for examining ROS levels.

### 2.6 Transwell Invasion Assay

In the upper chamber of the transwell plate, 2 × 10^4^ cells from all intervening groups (0, 10, 20, and 50 μM SchA) were introduced to each well. Pre‐coating of the Matrigel (BD Biosciences) onto the upper chamber surface occurred at 37°C for 1 h. The upper chamber received DMEM medium lacking FBS, whereas the lower chamber was supplemented with 500 μL of fresh medium comprising 10% FBS. Incubation of cells at 37°C with 5% CO_2_ was carried out for 24 h, succeeded by staining using 0.1% crystal violet solution at ambient temperature. Following 20 min, the invasion cells was counted with an optical microscope (Nikon).

### 2.7 Wound‐Healing Assay

All the intervening cells (0, 10, 20, and 50 μM SchA) were incubated within 6‐well plates at a density of 4 × 10^5^ cells to evaluate cell migration. A 200 μL pipette tip was employed to generate scratches in the cell monolayer. Following culture of cells in a complete medium at 37°C for 0 and 24 h, a light microscope (magnification, ×100; Nikon Corporation) was adopted for visualizing the plates and the wound closure. Image‐Pro Plus 6.0 software (Media Cybernetics, Inc.) was applied to analyze the cell migration rate.

### 2.8 Mitochondrial Membrane Potential (MMP) Detection

The damage to MMP in the cell was determined by the mitochondrial membrane potential detection kit (M8650, Solarbio, China). Dilution of the JC‐1 dye into the JC‐1 working solution was implemented obeying the manufacturer's handbook. The intervening cells (0, 10, 20, and 50 μM SchA) underwent resuspension utilizing the JC‐1 working solution (1 × 10^6^ cells/mL) and received 15 min of incubation at 37°C. Upon completion of staining, the cells underwent centrifugation at 240*g* for 3 min and were subsequently rinsed twice via the 1× buffer supplied in the kit. After being resuspended in 500 μL of the 1× buffer from the kit, the cells were analyzed with a CytoFLEX flow cytometer (Beckman Coulter, Inc.), with the FL4‐A: Y585‐PE channels and FL1‐A: B525‐FITC. Live cells with intact MMP were represented by red JC‐1 dye fluorescence whereas apoptotic or dead cells with damaged MMP by green JC‐1 monomer fluorescence. The ratio of fluorescence intensity of red (JC‐1 aggregate) to green (JC‐1 monomer) was utilized for evaluating MMP by FlowJo v10 software (BD Biosciences).

### 2.9 ATP, mtDNA, MDA, GSH, Fe^2+^ Assay

The levels of ATP, mtDNA, MDA, GSH, Fe^2+^ were detected by detection kit (Thermo Fisher Scientific, USA). Huh‐7 cell suspension was inoculated into a six‐well plate at a density of 2 × 10^5^ cells/well. After treating the cells with SchA (0, 10, 20, and 50 μM) at 37°C for 24 h, the medium was removed. Later, the cells were rinsed using pre‐cooled PBS, and 1 mL of 1× sample buffer was applied to lyse the cells in each well of the 6‐well plate. Then, the levels of ATP, mtDNA, MDA, GSH, and Fe^2+^ were tested as stated in the manuals of each kit.

### 2.10 Western Blotting

Western blotting analysis was conducted as previously described (Sun et al. 2024). Briefly, upon extraction of total protein from cells, the concentration detection was detected by BCA kit. The target protein was isolated through SDS‐PAGE, moved onto PVDF membrane, sealed via 5% skimmed milk in PBS, and then incubated using appropriate primary antibodies at 4°C overnight. The antibodies were listed as follows: GPX4 (ab262509, 1:1000), ACSL4 (ab155282, 1:10000), SLC7A11 (ab275537, 1:1000), AMPK (ab32047, 1:1000), p‐AMPK (ab133448, 1:1000), mTOR (ab134903, 1:10000), p‐mTOR (ab109268, 1:1000), S6K (ab308331, 1:500), p‐S6K (ab59208, 1:1000). Following washing, the membranes were incubated using horseradish peroxidase (HRP)‐labeled goat anti‐rabbit IgG (1:2000, 12‐348MSDS, Sigma) for 1 h at room temperature. Upon three times of washing utilizing PBS, the membranes were visualized through enhanced chemiluminescence (ECL) exposure solution, and analyzed via Image J software. The trial was given three repetitions.

### 2.11 Statistical Analysis

The mean ± standard deviation (SD) was adopted to present all data. Statistical analysis was finished by SPSS 22.0 (IBM, Armonk, NY, USA). Quantitative data underwent statistical significance evaluation utilizing one‐way ANOVA, then multiple comparisons were analyzed via Dunnett's test. *p* < 0.05 meant a statistical significance.

## 3 Results

### 3.1 SchA Inhibits the Activity of Hepatocellular Carcinoma Cells

To determine the impact of SchA on LO2 and HCC cells activity, we first examined its effect on cell viability and colony formation. Both cells were dealt with SchA at diverse concentrations (5, 10, 20, and 50 μM). The MTT assay revealed that the LO2 cell viability was not impacted by the increase in SchA concentration (*p* > 0.05), while the Huh7 cell viability was gradually inhibited with the up‐regulation of SchA concentration in contrast to the control group (*p* < 0.01) (Figure 1B). Consequently, we used the Huh7 cell line for subsequent experiments.

After treated with 10, 20, and 50 μM SchA at 24, 48, and 72 h, the cell viability of Huh7 cells was notably cut down relative to control group (*p* < 0.01) (Figure 1C). Moreover, the colony‐forming ability of Huh7 cells interfered with 10, 20, and 50 μM SchA was reduced dose‐dependently (*p* < 0.01) (Figure 1D). The above findings showed that SchA inhibited the activity of HCC cells.

### 3.2 SchA Facilitates the Hepatocellular Carcinoma Cell Apoptosis, and Inhibits Invasion and Migration

**FIGURE 2:**
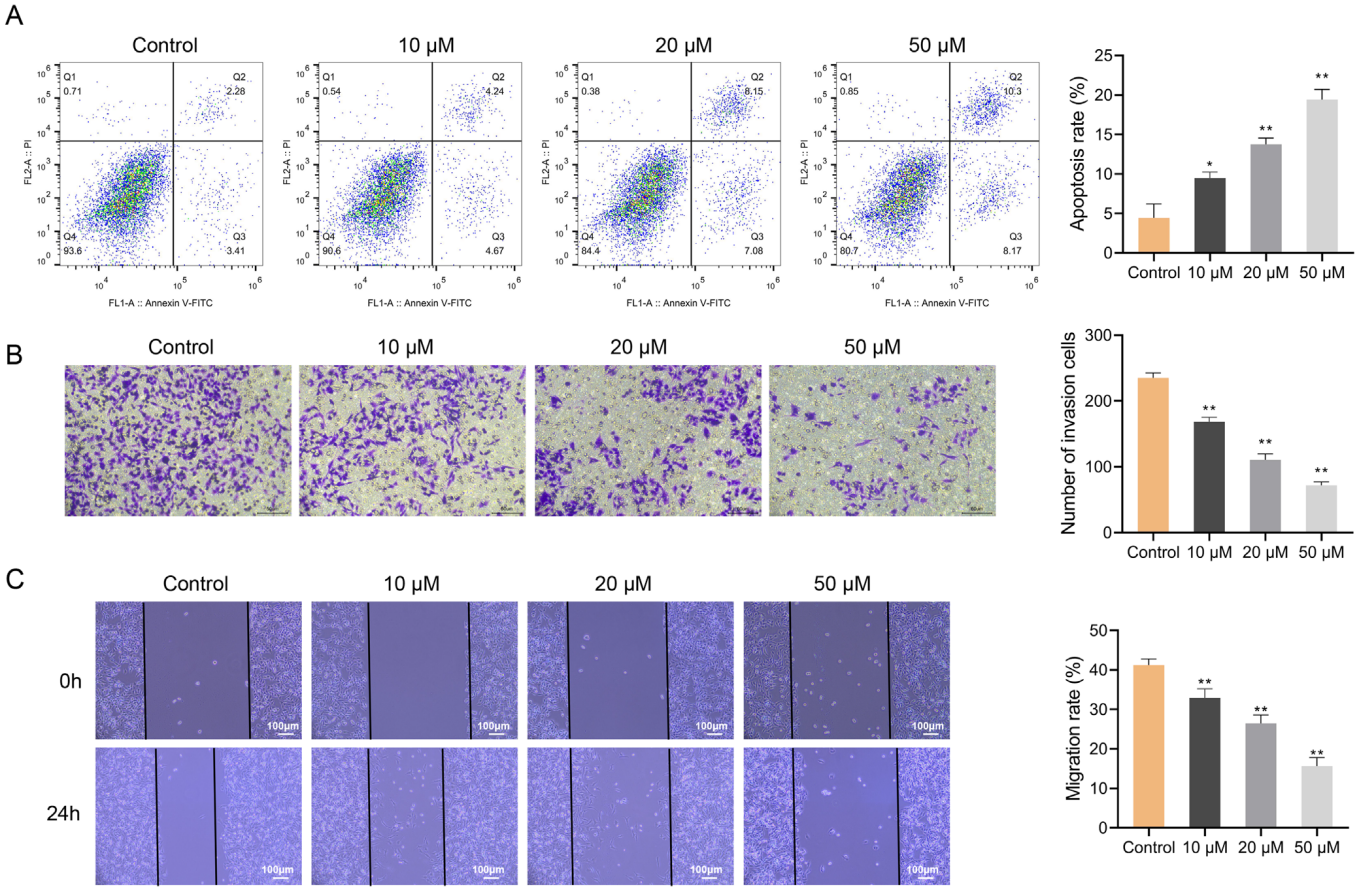
Schisandrin A promotes the apoptosis of hepatocellular carcinoma Huh7 cells, and inhibits invasion and migration. (A) The apoptosis of Huh7 cells treated with 0, 10, 20, and 50 μM Schisandrin A was detected by flow cytometry. (B) The invasion of Huh‐7 cells treated with Schisandrin A was detected by transwell assay. (C) The migration of Huh7 cells treated with Schisandrin A was detected by wound‐healing assay. *p < 0.05, **p < 0.01.

Following that, SchA's effects on the apoptosis, invasion and migration of Huh7 cells were estimated. The flow cytometry outcomes disclosed that increasing the dose of SchA strengthened the apoptosis of Huh7 cells (*p* < 0.05) (Figure 2A). Meanwhile, the transwell and wound‐healing test outcomes indicated that increasing the dose of SchA significantly blocked the invasion and migration of Huh7 cells in a dose‐dependent pattern (*p* < 0.01) (Figure 2B,C). The above evidence showed that SchA mediated apoptosis and inhibited the invasive and migratory capabilities of Huh7 cells.

### 3.3 SchA Induces Mitochondrial Dysfunction in Hepatocellular Carcinoma Cells

**FIGURE 3:**
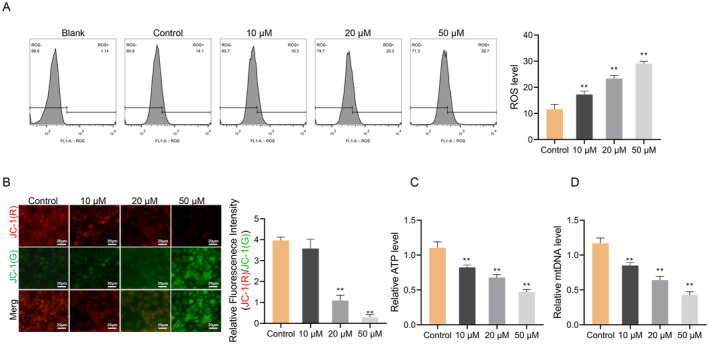
Schisandrin A induces mitochondrial dysfunction in hepatocellular carcinoma cell line Huh7. (A) Flow cytometry was used to detect ROS levels in Huh7 cells treated with 0, 10, 20, and 50 μM Schisandrin A. (B) Mitochondrial membrane potential changes in Huh7 treated with Schisandrin A were detected by JC‐1 assay. (C) ATP detection assay kit was used to detect the ATP levels in Huh7 cells treated with Schisandrin A. (D) mtDNA levels were quantitated via kit in Huh7 cells treated with 0, 10, 20, and 50 μM Schisandrin A. **p < 0.01.

We then explored the impact of SchA on oxidative stress and mitochondrial function in Huh7 cells. The intracellular ROS level was evaluated by DCFH‐DA probe. The flow cytometry results disclosed that as the SchA concentration increased, ROS levels also increased, particularly at concentrations of 20 and 50 μM (*p* < 0.01) (Figure 3A), which indicated that SchA up‐regulated the ROS level and destroyed the distribution of ROS in Huh7 cells.

Further, we assessed the impact of SchA on mitochondrial physiological function by examining mitochondrial membrane potential utilizing JC‐1 staining. The experimental findings demonstrated that as against the Control group, the red fluorescence/green fluorescence ratio in the 20 and 50 μM groups were markedly decreased (*p* < 0.01) (Figure 3B). In addition, the levels of ATP and mtDNA in Huh7 cells in 10, 20 and 50 μM groups declined markedly (*p* < 0.01) in a concentration‐dependent (Figure 3C,D). These results suggested that SchA induced mitochondrial dysfunction in HCC cells.

### 3.4 SchA Promotes the Ferroptosis of Hepatocellular Carcinoma Cells

**FIGURE 4:**
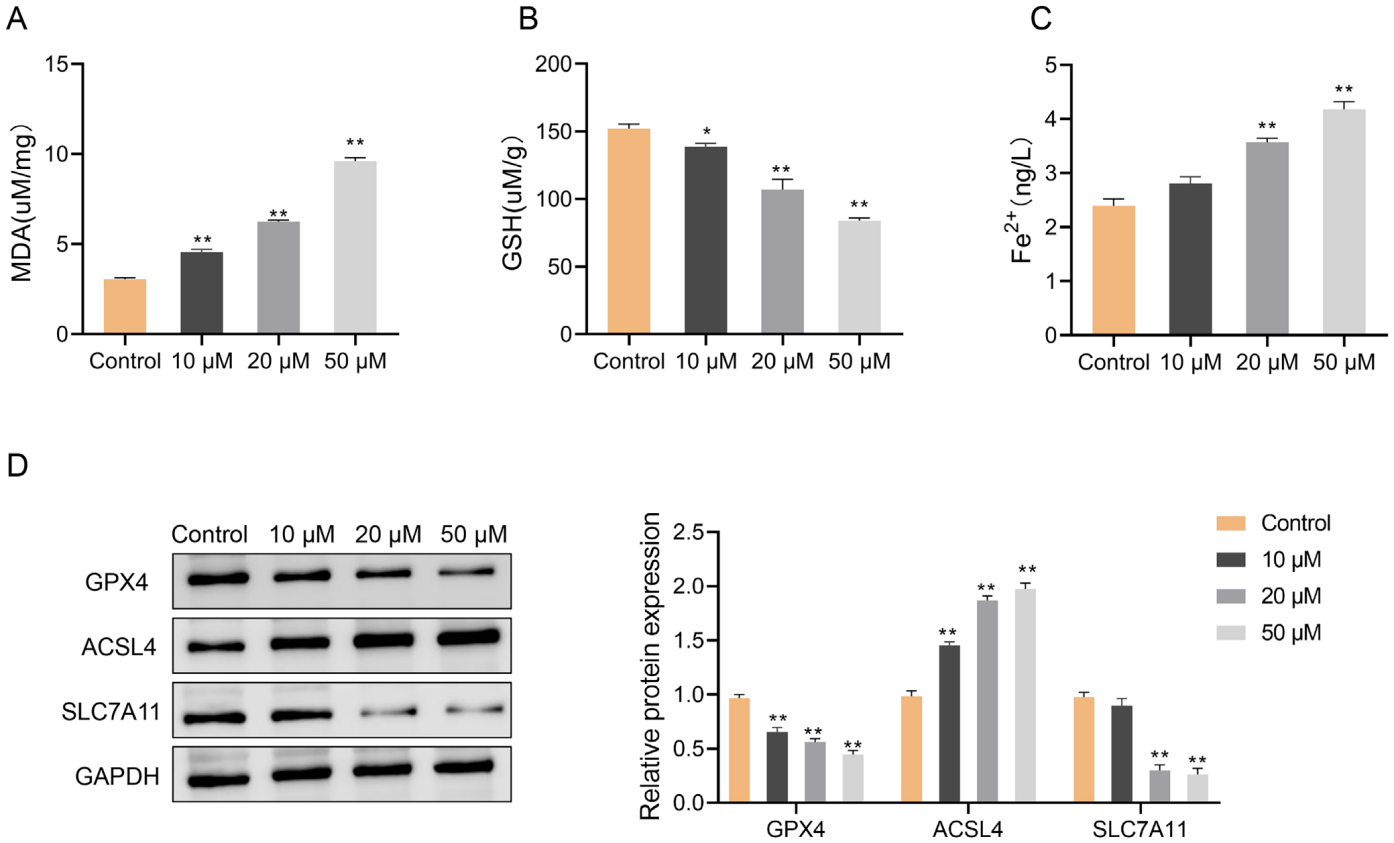
Schisandrin A promotes the ferroptosis of hepatocellular carcinoma Huh7 cells. (A, B) The MDA assay kit and GSH assay kit were used to detect the MDA levels (A) and GSH levels (B) in Huh7 cells treated with 0, 10, 20, and 50 μM Schisandrin A. (C) Analysis of Fe^2+^ iron content of Huh7 cells treated with Schisandrin A was examined, (D) Western blot was used to detect the expression of ferroptosis‐related proteins (GPX4, ACSL4, and SLC7A11) in Huh7 cells treated with Schisandrin A. *p < 0.05; **p < 0.01.

To investigate the role of SchA in ferroptosis, we analyzed oxidative stress markers and iron metabolism. The levels of MDA, GSH, and Fe^2+^ were identified with detection kits. Compared to the Control group, the MDA and Fe^2+^ levels in the 10, 20, and 50 μM groups were significantly raised, while GSH level significantly increased in the 20 and 50 μM groups (*p* < 0.05) (Figure 4A–C). Furthermore, the GPX4 and SLC7A11 protein expression levels in the 20 and 50 μM groups of Huh7 cells decreased significantly, while the ACSL4 protein level increased significantly (*p* < 0.01) (Figure 4D). These results suggested that SchA promoted oxidative stress and ferroptosis in HCC cells.

### 3.5 SchA Regulates AMPK/mTOR Pathway in Hepatocellular Carcinoma Cells

**FIGURE 5:**
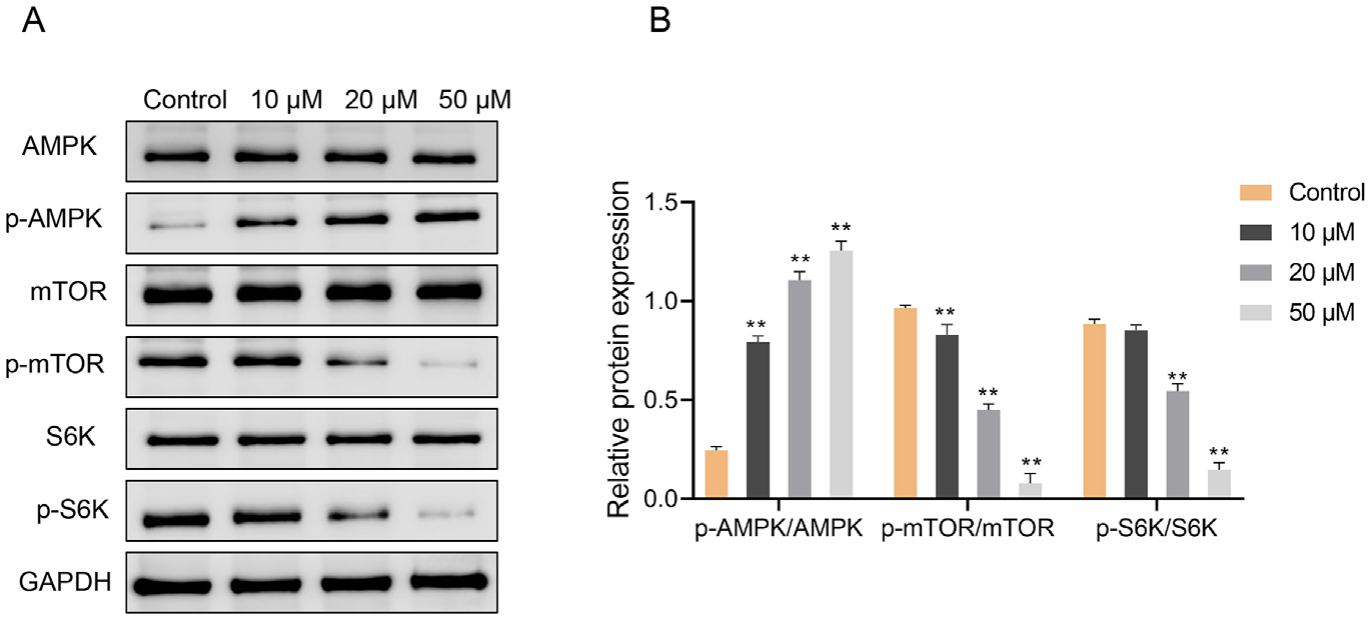
Schisandrin A regulates AMPK/mTOR pathway in hepatocellular carcinoma cell Huh7. (A, B) Western blot was used to detect the expression of AMPK, p‐AMPK, Mtor, p‐mTOR, S6K, and p‐S6K in Huh7 cells treated with 0, 10, 20, and 50 μM Schisandrin A. **p < 0.01.

Finally, we examined the effect of SchA on the AMPK/mTOR signaling pathway. Western blot analysis showed that treatment with 20 and 50 μM SchA significantly increased p‐AMPK level while decreasing the p‐mTOR and p‐S6K protein levels in Huh7 cells (*p* < 0.01) (Figure 5), which indicated that SchA could inhibit hepatocellular carcinoma by activating the AMPK signaling pathway and inhibiting S6K.

## 4 Discussion

Our research proposes an anti‐tumor role of SchA in HCC cells. The possible mechanism involves that SchA encourages the AMPK/mTOR pathway activation, triggers mitochondrial dysfunction, and enhances oxidative damage, ferroptosis and apoptosis of HCC cells, thereby inhibiting HCC cells' migration, proliferation and invasion and the HCC progression.

SchA is an antioxidant, which can play an anti‐tumor role by inhibiting oxidative stress (Zong et al. 2021). In this paper, SchA intervention caused the proliferation of HCC cells (Huh7 cells), the colony‐forming ability and the invasion rate of cell migration decreased significantly, and the apoptosis rate increased significantly. Moreover, SchA increased MDA content and decreased GSH level in Huh7 cells, which indicated that SchA promoted oxidative stress of HCC. In addition, the results of flow cytometry uncovered that SchA augmented the ROS level in HCC cells. The imbalance between the ROS generation and antioxidants is documented as the cause of oxidative stress (Marrocco, Altieri, and Peluso 2017). The augmentation of oxidative stress by ROS prompts cancer cell death (Wu, Ning, et al. 2020). Hence, our results indicate that the oxidative stress of HCC promoted by SchA is related to ROS activation.

Mitochondria are the main contributors of ROS, and mitochondrial dysfunction notably affects the progression of many tumors (Gao et al. 2020; Luo, Ma, and Lu 2020). Mitochondrial dysfunction primarily encompasses abnormalities in oxidative phosphorylation and aerobic glycolysis levels (Hsu, Tseng, and Lee 2016). The aerobic glycolysis activation level significantly furthers various tumors' proliferation and migration (Wu, Wu, et al. 2020). We found that SchA decreased the ATP production level of HCC cells. Furthermore, the flow cytometry outcomes exhibited that SchA significantly lessened the mitochondrial membrane potential expression in cells. In addition, mitochondria is also the main place for iron utilization, and mitochondrial dysfunction is likely to lead to abnormal iron accumulation secondary to ferroptosis (Battaglia et al. 2020). Recent studies have shown that ferroptosis plays a key role in the progression and treatment of many cancers. For instance, the research by Zou et al. (2022) provides a detailed analysis of how m6A modification regulates FGFR4 expression and its impact on ferroptosis in HER2‐positive breast cancer, offering new insights into potential therapeutic approaches for this challenging subtype of breast cancer. Additionally, Pei et al. (2023) provides comprehensive insights into the protective effects of baicalein against chemotherapy‐induced GI toxicity and highlights the therapeutic potential of targeting ferroptosis to mitigate side effects associated with cancer treatment. As a result, the iron content and ferritin expression in HCC cells were appraised. Specifically, SchA augmented Fe^2+^ and ACSL4 levels, and decreased GPX4 and SLC7A11 levels. These results confirmed that SchA caused mitochondrial dysfunction and promoted ferroptosis in HCC cells. Obviously, SchA‐caused ROS activation in HCC cells is related to mitochondrial dysfunction and ferroptosis, but the precise regulatory mechanism is still vague.

Studies have displayed that the increase of ROS will activate the expression of AMPK/mTOR pathway (Jiang et al. 2021). In this article, SchA intervention remarkably raised the p‐AMPK protein expression in cells, while the p‐mTOR and p‐S6K protein expression levels decreased remarkably. In the current research, SchA inhibited HCC by activating AMPK/mTOR pathway. Though there is no similar literature that directly proves that SchA activates AMPK pathway, our research displayed the vital role of SchA in regulating lipid and glucose metabolism (Naveed et al. 2018; Yu et al. 2021). AMPK is considered as a metabolic sensor to maintain metabolic homeostasis. As a consequence, we infer that SchA leads to mitochondrial dysfunction related to AMPK/mTOR pathway activation and promotes ferroptosis.

Despite initially identifying the crucial involvement of the ROS‐AMPK/mTOR pathway in the HCC regulation by SchA, this study has certain limitations. One limitation is the need for further exploration into how the AMPK/mTOR pathway influences mitochondrial energy metabolism and ferroptosis. Additionally, the absence of animal experiments presents another area for consideration. Next, we are going to research the specific regulation mechanism of this pathway combined with in vivo and in vitro experiments.

## 5 Conclusions

In summary, the current investigation shows that SchA may exert an anti‐cancer function in HCC through activating mitochondrial dysfunction and ferroptosis mediated by AMPK/mTOR pathway. Our research is anticipated to offer a novel theoretical foundation for applying SchA to treat HCC.

## Ethics Statement

The authors have nothing to report.

## Consent

The authors have nothing to report.

## Conflicts of Interest

The authors declare no conflicts of interest.

## Data Availability

The datasets used and/or analyzed during the current study are available from the corresponding author on reasonable request.
